# Differentiation of distal ureteral stones and pelvic phleboliths using a convolutional neural network

**DOI:** 10.1007/s00240-020-01180-z

**Published:** 2020-02-27

**Authors:** Johan Jendeberg, Per Thunberg, Mats Lidén

**Affiliations:** 1 Department of Radiology, Faculty of Medicine and Health, Örebro University Hospital, 70185 Örebro, Sweden; 2 Department of Medical Physics, Faculty of Medicine and Health, Örebro University, Örebro, Sweden

**Keywords:** Computed tomography, Ureteral calculi, Pelvic phlebolith, Deep learning, Convolutional neural networks

## Abstract

The objectives were to develop and validate a Convolutional Neural Network (CNN) using local features for differentiating distal ureteral stones from pelvic phleboliths, compare the CNN method with a semi-quantitative method and with radiologists’ assessments and to evaluate whether the assessment of a calcification and its local surroundings is sufficient for discriminating ureteral stones from pelvic phleboliths in non-contrast-enhanced CT (NECT). We retrospectively included 341 consecutive patients with acute renal colic and a ureteral stone on NECT showing either a distal ureteral stone, a phlebolith or both. A 2.5-dimensional CNN (2.5D-CNN) model was used, where perpendicular axial, coronal and sagittal images through each calcification were used as input data for the CNN. The CNN was trained on 384 calcifications, and evaluated on an unseen dataset of 50 stones and 50 phleboliths. The CNN was compared to the assessment by seven radiologists who reviewed a local 5 × 5 × 5 cm image stack surrounding each calcification, and to a semi-quantitative method using cut-off values based on the attenuation and volume of the calcifications. The CNN differentiated stones and phleboliths with a sensitivity, specificity and accuracy of 94%, 90% and 92% and an AUC of 0.95. This was similar to a majority vote accuracy of 93% and significantly higher (*p* = 0.03) than the mean radiologist accuracy of 86%. The semi-quantitative method accuracy was 49%. In conclusion, the CNN differentiated ureteral stones from phleboliths with higher accuracy than the mean of seven radiologists’ assessments using local features. However, more than local features are needed to reach optimal discrimination.

## Key points

**Table:** 

A Convolutional Neural Network for classifying pelvic calcifications was developed and validated.
The Convolutional Neural Network differentiated ureteral stones from phleboliths with higher accuracy than the mean assessment by seven radiologists.
More than local image features are needed to correctly classify pelvic calcifications

## Introduction

For more than 20 years, non-contrast enhanced computed tomography (NECT) has been the examination of choice for diagnosing ureteral stones. At detection, about two thirds (62–68%) of all ureteral stones are positioned in the lower part of the ureter (defined as overlying or below the sacroiliac joint) [1–3]. For a number of reasons, the assessment of this part of the ureter is a challenge even for an experienced radiologist. The lower ureters are located close to blood vessels, bowels and adnexa and, in lean patients in particular with a small amount of intra-abdominal fat, it may be impossible to separate those structures and identify a non-dilated distal ureter.

**Fig. 1:**
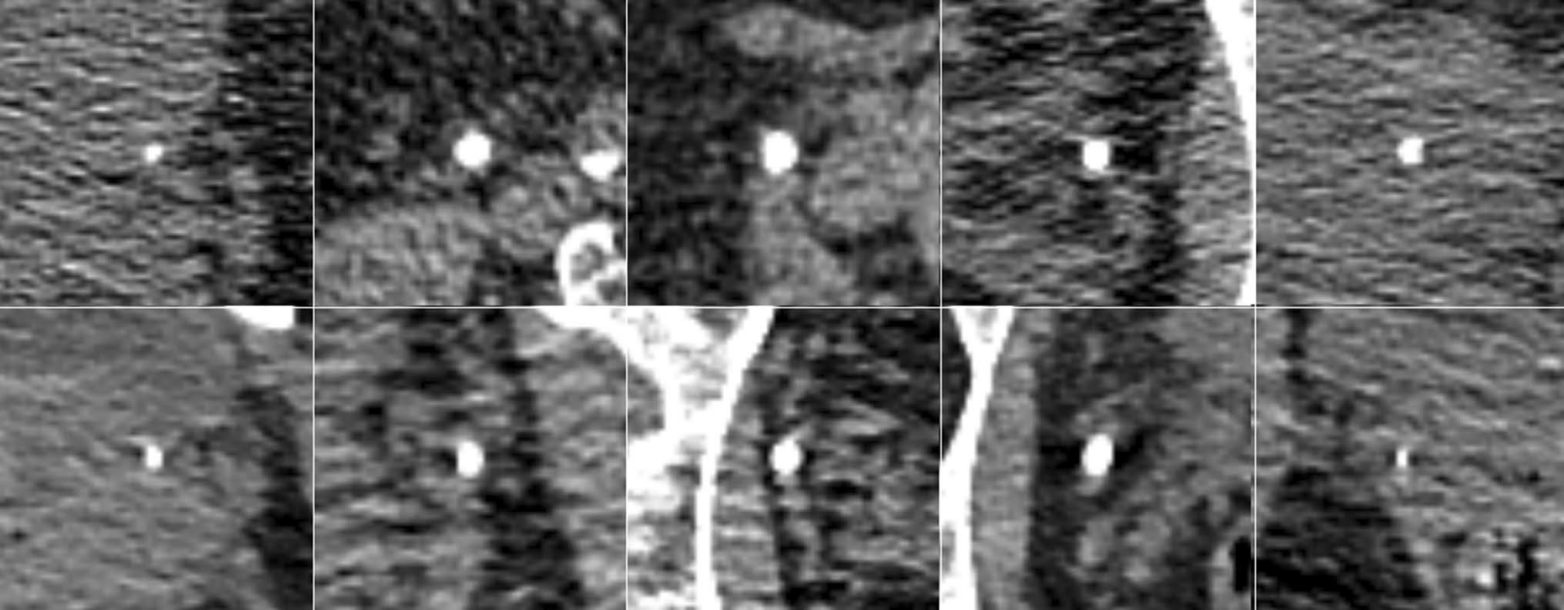
Examples of pelvic calcifications. *Upper row* Distal ureteral stones, *Lower row* Phleboliths

A frequent finding in the pelvis is phleboliths or wall calcifications of small veins. They have a prevalence of about 40% in the adult population [4], can be located close to the ureters and can be hard to distinguish from a distal stone. CT urography (CTU) to delineate the ureters is one method used to determine whether the calcification is a stone or a phlebolith. However, CTU has the disadvantage of increased radiation exposure and exposure to potentially nephrotoxic iodinized contrast media. Different approaches based on local features have been tried to distinguish urinary stones from phleboliths, such as the “soft tissue rim sign” [5–8] used to indicate a stone in the ureter and “the comet sign”, a central lucency or low attenuation [6, 9], used to indicate a phlebolith. In everyday practice, these signs are often insufficient for differential diagnosis, Fig. 1.

Another approach for differentiating stones from phleboliths is Computer-Aided Diagnosis (CAD). Differentiation using these methods is based on automatically or semi-automatically derived local image features of the calcifications. Recently, a semi-quantitative method was applied using cut-off values for the volume and attenuation of the calcification to discriminate stones from phleboliths [10]; while another method used image features that were fed into an artificial network [11]. Irrespective of the CAD method used, a key question for their development is whether the images of the calcification and its local surroundings can provide sufficient information for differentiation, or whether distant information, such as from visible upper ureters, is also needed.

Recent years have seen an enormous interest in artificial neural network (ANN)-based artificial intelligence (AI) in medical imaging [12, 13]. Briefly, an ANN is built from a simple mathematical nerve cell model, a neuron, which computes one output value from multiple input values. When a large number of neurons are arranged in inter-connected layers, the ANN can be optimized—trained—to predict outcomes based on the input of the first layer. A convolutional neural network (CNN) is an ANN with a specific arrangement in one or more layers called convolutional layers, which are especially suited for image analysis. A CNN can be fed with annotated images and learn to classify them through automatic iterative adjustments of the weighted neuron functions [14].

To the best of our knowledge, there are no previous studies that evaluate a CNN method for differentiating stones from phleboliths.

Therefore, with the overall aim of evaluating whether the assessment of a calcification and its surroundings is sufficient for discriminating distal ureteral stones from pelvic phleboliths in NECT, the objectives of this study were to: (1) Develop and validate a CNN method using local features for differentiating distal ureteral stones from pelvic phleboliths. (2) Compare the CNN method with a semi-quantitative method, and with radiologists’ assessments of the calcification and its local surroundings.

## Methods

The Regional Research Ethics Board approved this retrospective study and waived informed consent. We reviewed 1824 consecutive, acute NECT in patients referred in the period April 2012–September 2014 from the local Emergency Department with symptoms of suspected acute ureteral stone.

**Fig. 2:**
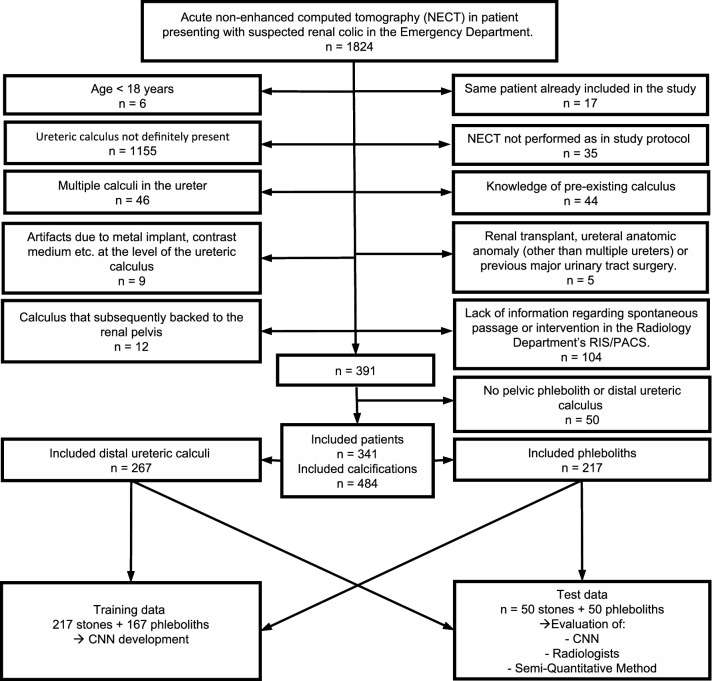
Flowchart of inclusion and exclusion criteria. *NECT *non-contrast enhanced computed tomography, *CNN *convolutional neural network

Inclusion and exclusion numbers and criteria are shown in Fig. 2. Sample size was defined according to a previous study using the same material [3] and therefore, no specific power analysis for the present study was performed.

From the retrospectively created data bank, 341 patients with a stone in the lower ureter (*n* = 267) and/or a pelvic phlebolith (*n* = 217) were included. We created a pseudonymized image stack of 1-mm slices around each calcification as a cube measuring 5 × 5 × 5 cm (fifty 1-mm images with a limited field of view of 5 × 5 cm) for use in the study.

The stone group consisted of 65 women and 202 men, with a mean age of 49 years (range 18–100). The phlebolith group included 49 women and 168 men; the mean age was 53 years (range 22–86). Average stone size (largest diameter) was 4.5 mm (range 2.0–11.8 mm) and average phlebolith size 4.5 mm (range 2.8–9.6 mm). 110 stones and 91 phleboliths were examined using a 40-detector row CT scanner (Brilliance, Philips Medical Systems Best, The Netherlands) with a low-dose NECT protocol for the urinary tract (120 kV, 70 mAs/slice, CTDI 4.9 mGy, 40 × 0.625 mm, standard filter [B], supine position). 157 stones and 126 phleboliths were examined using a 2 × 128-channel scanner (Somatom Definition Flash, Siemens, Erlangen, Germany) (120kVp, 70mAs/slice CTDI 4.7 mGy128 × 0.6 mm, filter B20f, B25f or I30f, supine position).

### Ground truth

At inclusion, one radiologist (with 12 years’ experience of reading abdominal CT) used the complete diagnostic NECT examination, as well as knowledge from all follow-up examinations until stone passage, potential previous examinations and the clinical information in the referral, to diagnose the ureteral stones. Ipsilateral hydronephrosis, hydroureter, perinephric and periureteral stranding and the clinical information, as site of pain, were used for guiding to a distal ureteral stone, which had to be clearly visible in the distal ureter to be included.

When a pelvic phlebolith was present, the one most likely to be mistaken for a distal ureteral stone was included in the study. This assessment was subjective, but based on the size and nearness to the distal part of one the ureters. When necessary, several prior and subsequent examinations were used to define a phlebolith as such.

If there was doubt of the classification into a distal ureteral stone or a pelvic phlebolith, the calcification was not included.

### Test dataset

As illustrated in the flowchart (Fig. 2), the stacks with calcifications were randomly split into two separate datasets. We created one smaller dataset containing 50 stones and 50 phleboliths from the included calcifications. This dataset was not used for training the neural network, but served as a validation dataset when evaluating the three methods for their ability to differentiate phleboliths from stones.

### Convolutional neural network (2.5D-CNN)

A convolutional neural network was developed and trained using the training dataset comprised of the remaining 217 ureteral stones and 167 phleboliths. A 2.5-dimensional CNN (2.5D-CNN) model was used, where three perpendicular 2D images (axial, coronal and sagittal) were created having each calcification voxel as intercept. The image triplets for all voxels were used as input data for the CNN [15]. Each calcification was segmented using a threshold of 250 HU, and any hole in the segmented volume was filled using a morphologic operation.

The training data were augmented by mirroring in the left–right plane, where the anatomic differences are small.

Using each voxel location as a separate training example, each calcification contributed from a few to more than one thousand training examples, depending on its size. In total, the 384 stones and phleboliths generated 38 068 training examples, i.e., image triplets.

**Fig. 3:**
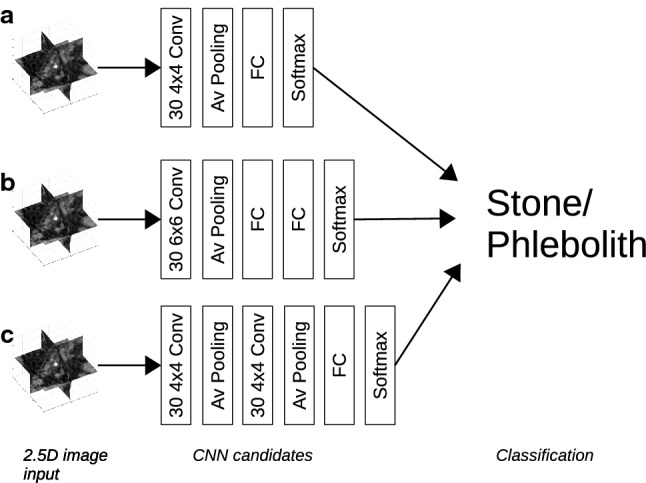
Schematic architecture of the three 2.5D convolutional network (2.5D-CNN) candidates validated on the test dataset. *Conv *convolutional layer, *Av *average pooling layer**,** *FC *fully connected layer

Several different 2.5D-CNN architectures were tested on the training data and three different network candidates were selected, see Fig. 3. After selection, the three candidate networks were trained on the full training cohort.

For classification on the unseen test set, each calcification was segmented and 2.5D images generated similarly as in the training set, but without mirroring. The classification was performed on each voxel location in the calcification, and the output for each calcification was computed as the average probability output of the softmax classification layer. A cut-off of 0.5 on the final score was used for classification as stone or phlebolith.

### Radiologist assessments

Seven radiologists, with 10–28 years of experience reading abdominal CTs independently reviewed the one hundred 5 × 5 × 5 cm 1-mm stacks in the test data, using standard PACS (IDS7 Sectra AB, Linköping, Sweden). The readers were allowed to use all available features in the system, such as multiplanar reformats (MPR), zoom, attenuation and size measurements. The readers were blinded to the result of the reference standard, clinical information and distant image information (such as hydronephrosis, perirenal fat stranding etc.).

### Semi-quantitative method using attenuation and volume

Following the protocol for a recently published study [10], classification of distal ureteral stones and phleboliths was performed based on the cut-off values below:

Attenuation > 643HU →Ureteral stone.

Attenuation < 643HU →Phlebolith.

Volume > 171mm^3^ →Ureteral stone.

Volume < 171mm^3^ →Phlebolith.

The same window settings (W300/L40) and slice thickness of 5 mm as in the original study were used for the measurements for the semi-quantitative method. The 5-mm slices were created as MPR based on 1-mm images. The volume of the calcification was calculated with the ellipsoid formula used in the original article, W × L × H × Pi/6, where W, L and H are the lengths of the three principal axes of the ellipsoid. The mean attenuation was measured in the center of the calcification using the in PACS integrated tool for attenuation measurement, the region of interest (ROI) circle.

The size measurements of the calcifications were performed manually by a radiologist with 14 years’ experience reviewing abdominal CTs. He was blinded to the results of the reference test, distant image information and clinical information and to the results of the assessments by the seven radiologists.

### Statistics

Statistical analysis was performed using IBM SPSS for Mac OS v24.0.0.0 (SPSS Inc. Chicago, Il. USA) and Matlab R2018b (The Mathworks Inc, Natick, Mass, USA). The neural networks were developed using Matlab Deep Learning Toolbox.

Sensitivity, specificity and accuracy with 95% confidence intervals (CI) using binomial distribution were calculated for the neural network and for the radiologists’ classifications compared to the reference standard. The average accuracy for the seven readers was calculated as well as the “vote majority”, defined as the assessment of each calcification chosen by the majority of the readers (≥ 4).

The statistical significance of the difference between the CNN and the mean accuracy of the readers was tested using the one sample *t* test.

In the semi-quantitative method, the differences in volume and attenuation between stones and phleboliths were not normally distributed and were, therefore, analyzed using the Mann–Whitney *U*-test. The scatter plot was visually analyzed for alternative cut-off values for the semi-quantitative method. Where applicable, the area under the Receiver Operating Characteristic (ROC) curve (AUC) was computed.

## Results

### Validation on test dataset

Neither the radiologists, nor the 2.5D-CNN, nor the semi-quantitative method could fully differentiate between the pelvic calcifications in the test dataset. Cross tabulations for the three methods against the reference standard are shown in Table 1.

**Table 1:** Cross tabulations and accuracy with 95% CI for three methods for differentiation of 50 distal ureteral stones and 50 pelvic phleboliths using only local features

	Reference standard			
		Phlebolith	Stone	Total
(a) 2.5D-CNN Prediction Stone vs phlebolith				
2.5D-CNN	Phlebolith	45	3	48
	Stone	5	47	52
	Total	50	50	100
Accuracy 92% (95% CI 85–97%)				
(b) Cooperation all readers Prediction Stone vs phlebolith				
Majority of the readers assessments	Phlebolith	49	6	55
	Stone	1	44	45
	Total	50	50	100
Accuracy 93% (95% CI 86–97%)				
(c) Median reader assessment				
Median reader	Phlebolith	44	5	49
	Stone	6	45	51
	Total	50	50	100
Accuracy 89% (95% CI 81–94%)				
(d) Semi-quantitative method: ≤ 643HU and ≤ 173mm³ →Phlebolith				
Quantitative prediction volume + HU	Phlebolith	38	39	77
	Stone	12	11	23
	Total	50	50	100
Accuracy 49% (95% CI 39–59%)				

Reference standard = Calcification assessment by a radiologist using the complete NECT examination as well as knowledge from follow-up examinations until stone passage and clinical information

(a) Convolutional neural network (2.5D-CNN) using the information from perpendicular axial, coronal and sagittal images intersecting each voxel of the calcification

(b) Majority vote of seven radiologists using only a 5 × 5 × 5cm large 1-mm slice stack surrounding the calcification

(c) Median radiologist assessment using only a 5 × 5 × 5cm large 1-mm slice stack surrounding the calcification

(d) Semi-quantitative method using only the volume (mm^3^) and attenuation (HU) of each calcification in a 5-mm slice stack

### 2.5D-CNN

There were only small differences in classification accuracy for the pelvic calcifications between the three candidate networks.

The accuracy on the unseen test set was 93%, 90% and 92% and the AUC was 0.93, 0.93 and 0.95 for the three 2.5D-CNN candidates, respectively. For the further analysis, the third network candidate (Fig. 3c), with two convolutional layers, was selected. The sensitivity, specificity and accuracy for the third 2.5D-CNN candidate were 94% (95% CI 87–98%), 90% (95% CI 82–95%) and 92% (95% CI 85–97%), respectively.

### Radiologist assessment

**Fig. 4:**
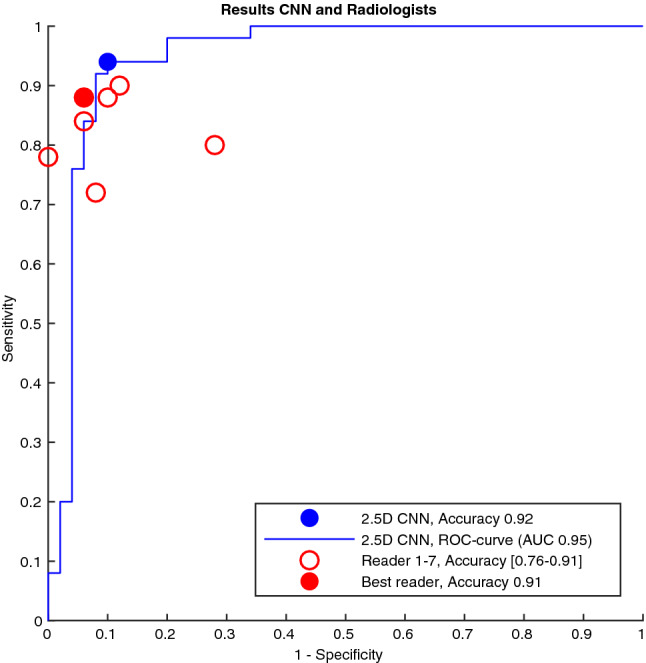
Reciever operator characteristics (ROC) curve for a convolutional neural network candidate compared to the accuracy of the assessements by seven radiologists

The average accuracy for the seven radiologists’ classifications was 86% (range 76–91%), which was significantly lower (*p* = 0.03) than the accuracy of the third 2.5D-CNN. Using a majority vote among the readers (classification of each calcification chosen by ≥ 4 readers) for the assessments of the calcifications, the accuracy rose to 93% (95% CI 86–97%), sensitivity 88% (95% CI 80–94%) and specificity 98% (95% CI 93–100%). The accuracy of the reader with the median result was 89% (95% CI 81–94%). The ROC curve for the third 2.5D-CNN compared to the radiologists' results is shown in Fig. 4.

### Semi-quantitative method

There was no significant difference in the median volume (*p* = 0.70) or the median attenuation (*p* = 0.29) between the lower ureteral stones and pelvic phleboliths.

**Fig. 5:**
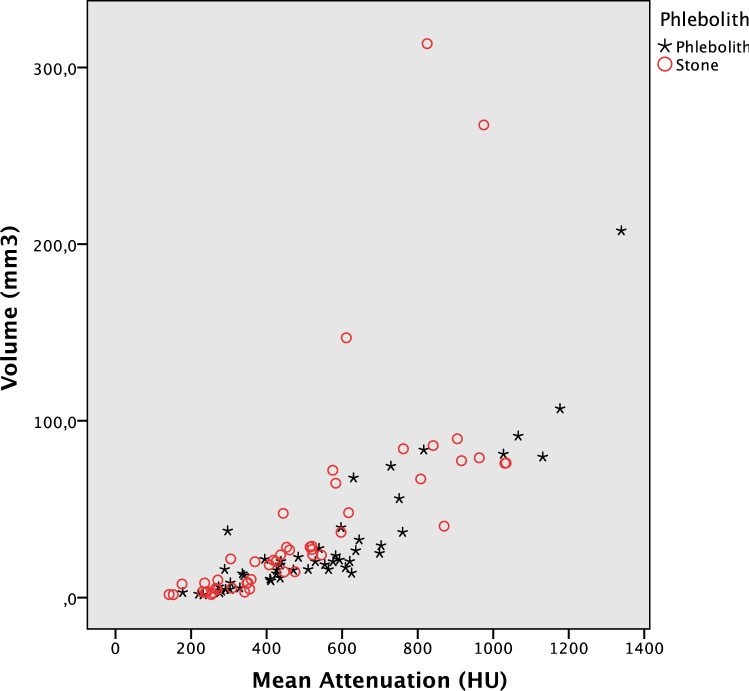
Scatter plot with manual measurements of volume and attenuation. *X*-axis: Mean attenuation (mean HU), *Y*-axis: Volume (mm^3^), *Red circles* Distal ureteral stones. *Black stars* Phleboliths

The AUC for classifying a pelvic calcification as a stone or phlebolith using the semi-quantitative method was 0.56 (95% CI 0.45–0.68) and 0.52 (95% CI 0.41–0.64) for the attenuation and volume measured on 5-mm images, respectively. Use of the proposed cut-off values of 643HU and 171mm^3^ did not enable differentiation between a stone and a phlebolith, see Fig. 5 and Table 1 d.

The scatter plot did not reveal an alternative cut-off suitable for differentiation between a ureteral stone and phlebolith.

## Discussion

In this study, we showed that a convolutional neural network (2.5D-CNN) that had been trained on a dataset of 384 pelvic calcifications, using only local image features, could classify calcifications in an unseen test set into lower ureteral stones and phleboliths with an accuracy of more than 90%. This result was similar to the majority vote among seven radiologists, also using only local image features, having a pooled accuracy of 93%,  and significantly better than the mean of the same radiologists’ results.

In recent years, a large number of studies have been performed using machine learning in radiology in many different applications including neuroimaging and imaging of the chest and abdomen, where the main interest has been towards oncology imaging and anatomy [12]. In two previous studies, CNNs have been used for the detection of ureteral stones [16, 17]. We are not aware of any study previously published on the differentiation between lower ureteral stones and pelvic phleboliths on NECT using CNN.

In 2010, Lee et al. [11] created an artificial neural network (ANN) that used combinations of various shape and internal texture parameters of 112 calcifications to classify them into ureteral stones or vascular calcifications. They reached an AUC of 0.85 for the shape parameters and 0.88 for texture parameters. However, the same study group was used for the ANN development and for validation, and their true AUC on a separate test set must be presumed to be lower.

To the best of our knowledge, the accuracy of 92% and an AUC of 0.95 on the unseen test set for the third candidate 2.5D-CNN in our study is the best published result for this computer-aided diagnosis (CAD) application. These results are promising for the development of CAD for pelvic calcifications but, to be clinically useful, greater accuracy is necessary than that expected from an experienced radiologist using the information contained in the complete NECT. It is also of major importance that ureteral stones are not falsely classified as phleboliths, which was the case in three out of 50 stones (6%) in our test data set.

Neither the individual radiologists, nor the majority vote among the radiologists, nor the 2.5D-CNN could completely differentiate between stones and phleboliths using only local features in a 5 × 5 × 5 cm large cube surrounding the calcifications. This finding strongly suggests that local features are not sufficient for this discrimination and that future CNN models should contain distant information, such as all parts of the urinary tract and its closest surroundings for improved performance [17]. In daily practice, the radiologists assess various information such as hydronephrosis, hydroureter, perirenal or periureteral fat stranding, and information in the referral about ipsi- or contralateral symptoms together with the regional image information close to the calcification. Analogous to the performance of human readers, it is reasonable to believe that inclusion of more information sources in the ANN could improve the machine learning performance.

Earlier studies on visual local features and signs have shown divergent results, with mostly high specificity as, for example, 92–100% [6, 9] for the rim sign indicating a ureteral stone and up to 100% for the comet sign [5, 7–9] indicating a phlebolith. The sensitivity has been lower, 50–77% and 21–65%, respectively, and the authors have recommended the use of distant and clinical information along with the local features for the assessment of pelvic calcifications. In a study of Rochester Guest et al. the rim sign was accompanied by distant features of obstruction in all cases but one and the same study also found a pseudo-comet sign in some of the ureteral stones [18]. The central lucency that was used to differentiate phleboliths from stones on plain radiographs has shown to be present only occasionally (1–9%) on NECT [9, 19].

The ability of a recently published semi-quantitative method that used the attenuation and volume of the calcifications to differentiate between stone and phlebolith could not be confirmed in our study. The volume criterion in the semi-quantitative method is highly sensitive for inclusion criteria [20] and, similar to us, Bell et al. [9] did not find a significant difference in size between distal ureteral stones and phleboliths. In our study, we included the phlebolith most likely to be mistaken for a ureteral stone, as we considered that the radiologist or urologist would need automated assistance with this calcification. This might have contributed to the lack of difference in volume and attenuation between ureteral stones and phleboliths in our material.

Our study has limitations. Even though we used the largest dataset with labeled pelvic calcifications that has been used for machine learning so far, more training examples might have increased the accuracy of the CNN. A generalization of the CNN method would also require training examples from a wide range of scanners with different protocol parameters.

In regard to the inclusion of phleboliths, only proven phleboliths (proven with the help of consecutive examinations, distance NECT information, etc.) were included. This might have resulted in a bias towards a better accuracy than achievable in daily practice. We endeavored to minimize this bias by selecting the phlebolith most likely to be mistaken for a stone. Inclusion was performed retrospectively and only a very small number of the patients underwent ureteroscopy, which could, therefore, not be used for defining ground truth. However, we do not regard the lack of ureteroscopy as reference standard to be a major limitation. Ureteroscopy has two disadvantages compared to multiple radiologic follow-up examinations. Firstly, it cannot confirm the presence of a phlebolith, merely the presence of a ureteral stone. Secondly, spontaneous stone passage in the interval between the CT examination and the ureteroscopy could lead to false-negative results of the ureteroscopy.

In conclusion, we demonstrated that a 2.5D-CNN could differentiate ureteral stones from phleboliths with a 92% accuracy, which is higher than the mean accuracy of assessment by seven radiologists. This finding suggests that AI can become a valuable tool for ureteral stone imaging. In contrast, the semi-quantitative method had a significantly lower accuracy. Importantly, neither the CNN, nor the majority vote by seven trained readers was entirely accurate, which suggests that more than local image features, such as information from the complete CT examination and clinical information, is needed for the discrimination between distal ureteral stones and pelvic phleboliths.

## Abbreviations

NECT: Non-contrast-enhanced computed tomography
CTU: CT urography
CNN: Convolutional neural network
2.5D-CNN: 2.5-Dimensional CNN including axial, coronal and sagittal images
CAD: Computer-aided diagnosis
ANN: Artificial neural network
AI: Artificial intelligence
FC layer: Fully connected layer
PACS: Picture archiving and communication system
MPR: Multiplanar reconstructions
CI: Confidence interval
ROC: Receiver operating characteristic
AUC: Area under the curve
ROI: Region of interest
